# An assessment of remotely sensed environmental variables on Dengue epidemiology in Central India

**DOI:** 10.1371/journal.pntd.0010859

**Published:** 2022-10-17

**Authors:** Devojit Kumar Sarma, Manoj Kumar, Praveen Balabaskaran Nina, Karuppusamy Balasubramani, Malay Pramanik, Rintu Kutum, Swasti Shubham, Deepanker Das, Manoj Kumawat, Vinod Verma, Jigyasa Dhurve, Sekar Leo George, Alangar Balasundreshwaran, Anil Prakash, Rajnarayan R. Tiwari

**Affiliations:** 1 ICMR- National Institute for Research in Environmental Health, Bhopal Bypass Road, Bhouri, Bhopal, Madhya Pradesh, India; 2 Department of Epidemiology and Public Health, Central University of Tamil Nadu, Thiruvarur, Tamil Nadu, India; 3 Department of Public Health and Community Medicine, Central University of Kerala, Kasaragod, Kerala, India; 4 Department of Geography, School of Earth Sciences, Central University of Tamil Nadu, Thiruvarur, Tamil Nadu, India; 5 Urban Innovation and Sustainability Program, Department of Development and Sustainability, Asian Institute of Technology, Klong Luang, Pathumthani, Thailand; 6 Department of Computer Science, Ashoka University, Sonipat, Haryana, India; 7 Trivedi School of Biosciences, Ashoka University; 8 Stem Cell Research Centre, Department of Hematology, Sanjay Gandhi Post-Graduate Institute of Medical Sciences, Lucknow, Uttar Pradesh, India; University of California Davis, UNITED STATES

## Abstract

In recent decades, dengue has been expanding rapidly in the tropical cities. Even though environmental factors and landscape features profoundly impact dengue vector abundance and disease epidemiology, significant gaps exist in understanding the role of local environmental heterogeneity on dengue epidemiology in India. In this study, we assessed the role of remotely sensed climatic factors (rainfall, temperature and humidity) and landscape variables (land use pattern, vegetation and built up density) on dengue incidence (2012–2019) in Bhopal city, Central India. Dengue hotspots in the city were assessed through geographical information system based spatial statistics. Dengue incidence increased from 0.59 cases in 2012 to 9.11 cases in 2019 per 10,000 inhabitants, and wards located in Southern Bhopal were found to be dengue hotspots. Distributed lag non-linear model combined with quasi Poisson regression was used to assess the exposure-response association, relative risk (RR), and delayed effects of environmental factors on dengue incidence. The analysis revealed a non-linear relationship between meteorological variables and dengue cases. The model shows that the risk of dengue cases increases with increasing mean temperature, rainfall and absolute humidity. The highest RR of dengue cases (~2.0) was observed for absolute humidity ≥60 g/m^3^ with a 5–15 week lag. Rapid urbanization assessed by an increase in the built-up area (a 9.1% increase in 2020 compared to 2014) could also be a key factor driving dengue incidence in Bhopal city. The study sheds important insight into the synergistic effects of both the landscape and climatic factors on the transmission dynamics of dengue. Furthermore, the study provides key baseline information on the climatic variables that can be used in the micro-level dengue prediction models in Bhopal and other cities with similar climatic conditions.

## Author summary

Dengue, a viral disease transmitted by infected *Aedes* mosquitoes, is a major public health concern globally. In addition to its increased incidence in recent years, dengue is also spreading to new geographical regions. Local environmental factors are known to modify the mosquito vector density that directly impacts dengue virus transmission. Understanding the influence of environmental factors (meteorological conditions and landscape features) on dengue epidemiology in local settings is important for focused dengue intervention. Here, by utilizing dengue incidence and remotely sensed environmental data from 2012–2019, we have assessed the role of environmental factors in driving dengue virus transmission in the city of Bhopal in Central India. During the study period, a 14.5 fold increase in dengue incidence was observed in Bhopal city, which is way higher than the 2.3 fold increase reported at the national level. The risk of dengue virus transmission was higher with higher temperature and absolute humidity. An increase in built-up area, a proxy for urbanization, was found to be another predictor of increased dengue incidence in Bhopal. These findings can provide a stepping-stone for the development of dengue prediction models and the identification of dengue hotspots in order to improve vector control of this disease in cities with similar environmental conditions.

## Introduction

Dengue is a serious mosquito-borne viral infection that affects more than half of the world’s population. Globally, a 30 fold increase in dengue cases has been observed in the last five decades [1,2]. In recent years, the highest dengue incidence has been reported in South Asia, and is followed by Southeast Asia and tropical Latin America [3]. Climate change and urbanization are expected to facilitate the geographical expansion of dengue further, leading to a severe public health burden [4]. With an estimated annual global morbidity of >104 million and >40,000 deaths in 2020, dengue attributed Disability Adjusted Life Years have increased by ~ 107% in 195 countries [3,5,6].

Ever since the first recorded dengue outbreak in 1963 from Calcutta city in India [7], many outbreaks at various time periods have been reported from different parts of the country [8,9]. Dengue virus is primarily transmitted by *Aedes aegypti* and *Ae*. *albopictus* mosquitoes. India has witnessed an increase in dengue cases since 2001 with all four serotypes [2,10,11]. Initially, dengue was more predominant in urban and semi-urban areas in India. However, dengue has recently spread to rural areas, and could affect the entire Indian population [11–13]. During 2010–2014, the dengue incidence in India increased by more than 5 folds (34.81 per million) as compared to 1998–2009 (6.34 per million) [2]. Even though dengue is a notifiable disease in India, many studies suggest it is grossly under-reported, and the actual burden of dengue in the country is poorly quantified [14,15]. In 2019, a total of 157,315 cases of dengue and 166 deaths related to dengue were reported in India [16]. On the other hand, the Global Burden of Disease 2017 study reported an age-standardized dengue incidence rate of 4072.9 per 100,000 inhabitants in India which is one of the highest globally [3]. A nationwide serosurvey carried out in 2017–18 estimated 48.7% of the Indian population were seropositive for dengue [15].

Dengue virus transmission is highly influenced by meteorological factors such as temperature, rainfall and relative humidity (RH) [17]. Warm temperatures provide favorable conditions for the growth and development of vector mosquitoes and affect the length of the gonotrophic cycle, the extrinsic incubation period (EIP) of the virus within *Aedes* mosquitoes, and the basic reproduction number (R_0_), thereby influencing the transmission dynamics of the disease [18–26]. The diurnal temperature range also influences the infectivity of the dengue virus in *Ae*. *aegypti* [27]. Similarly, rainfall provides habitat for mosquito breeding and increases the abundance of vector mosquitoes [28]. High humidity also favors the survival and biting of adult vector mosquitoes [29,30].

Dengue transmission is also influenced by anthropogenic and environmental factors such as the degree of urbanization, human mobility, land use change, and vegetation [31–33]. An increase in urbanization has led to heterogeneous socio-economic and environmental conditions that facilitate dengue transmission [34]. Increased population density along with poor socio-economic conditions and distressed environmental hygiene provide a suitable environment for vector breeding and increased human-vector contact and dengue transmission [34–38]. In the past few decades, India has been undergoing rapid urbanization, and as of 2018, 34% of India’s population were living in urban areas compared to 11% in 1901 [39]. Rapid urbanization with concomitant changes in vegetation cover and land use types will result in urban heat islands (warmer temperatures than nearby rural areas) that can significantly impact urban micro-climates [40]. This in turn can modify the local vector populations, increase host-vector contact and alter the transmission dynamics of dengue virus [41–44].

Even though most Indian cities experience a tropical or subtropical climate, local variations in topography and land use affect micro-climatic conditions and local dengue epidemiology. This environmental heterogeneity has also been documented by differences in EIP, an important entomological parameter to measure dengue epidemiology in different parts of the country [2]. Due to this environmental heterogeneity, a common dengue disease model for early warning and outbreak prediction may not be applicable to the entire country [2,45]. An elegant study by Kakarla et al. [45] modeled the lagged effects of meteorological variables on dengue incidence in India. However, significant gaps persist, especially on our understanding on the role of local climatic and landscape features on dengue incidence at the city level. To understand the role of local climatic factors and urbanization on dengue incidence (2012–19) in Bhopal city in Central India, relevant remotely sensed datasets were analyzed and the impact of environmental variables on dengue incidence is detailed. The outcome of this study will greatly help to understand, predict, and control dengue transmission in endemic areas with similar eco-climatic conditions.

## Materials and methods

### Study area

**Fig 1:**
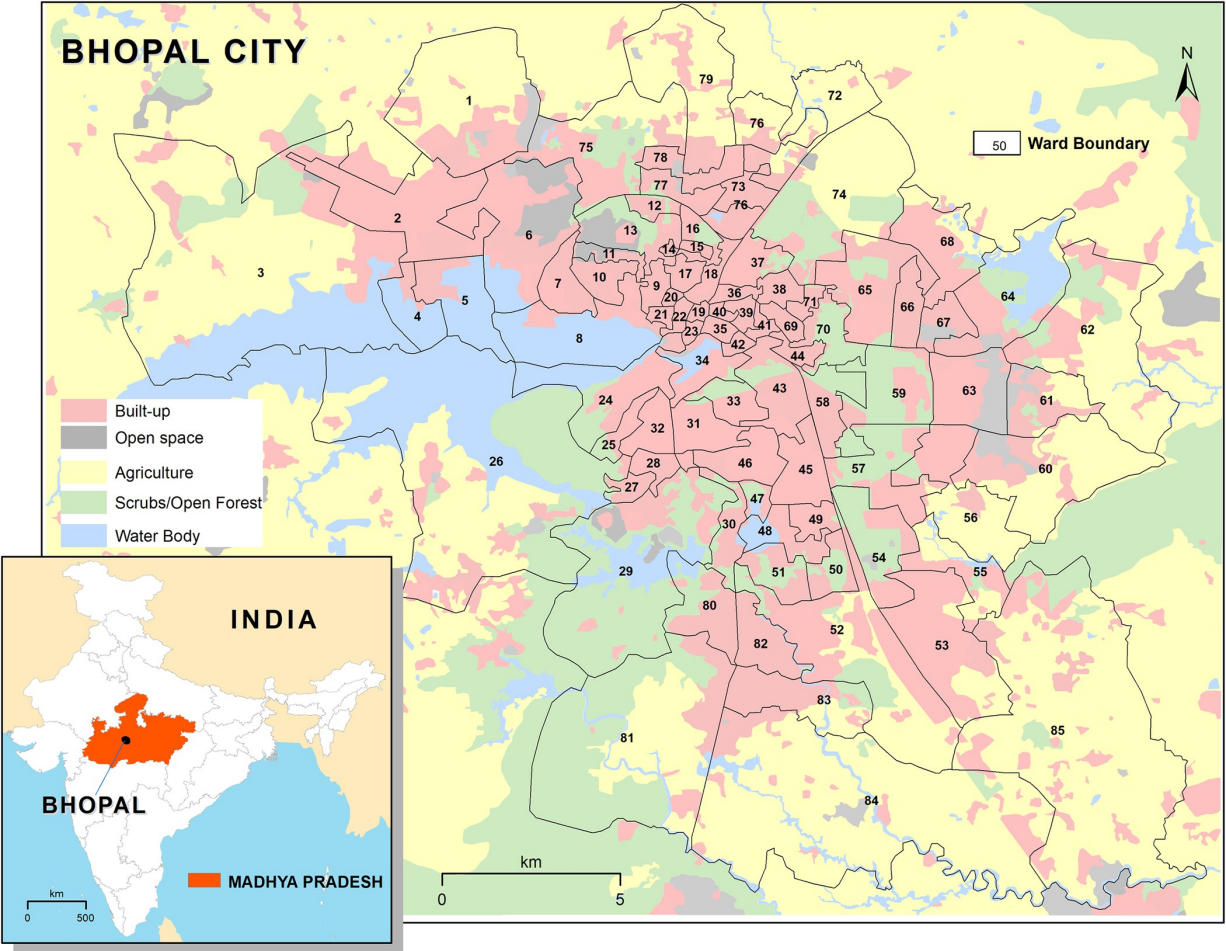
Location map and administrative boundaries of Bhopal city, Madhya Pradesh, India (Source of shapefile: http://projects.datameet.org/Municipal_Spatial_Data/).

Bhopal (23.25°N, 77.40°E), the capital city of Madhya Pradesh state in Central India, is located in the sub-tropical climate zone. It encompasses an area of 285.9 km^2^ with an average altitude of 500m above mean sea level and possesses dry mixed deciduous forest cover. The city has a total population of 1.9 million [46], with a population density of 3,887 persons/km^2^. The city is divided into 85 wards, which act as administrative and health service delivery units (Fig 1). The topography of Bhopal city is uneven and characterized by a number of small hillocks and large water bodies. The city is surrounded by dense to open scrub forests, and the major land use types are sparse to high-density built-ups, water bodies, agricultural crop lands, and a range of fallow/waste lands. The climate is characterized by dry winter, hot summer, and humid monsoon seasons with an annual average temperature of 25°C (ranges from 15°C to 40°C), and rainfall of ~ 1200 mm. The summer season starts in March, peaks in May (exceeds 40°C) and continues till June. The monsoon season starts in late June and ends in late September. The winter season (November—February) peaks in January when the night temperature may drop close to the freezing point. The average RH ranges from 20% (summer) to 90% (monsoon).

### Data collection

#### Collection of epidemiological data

In India, dengue is diagnosed primarily based on clinical manifestations (high fever, headache, retro-orbital pain, myalgia, arthralgia, rash and hemorrhagic manifestations) and laboratory diagnosis [47]. Clinically suspected dengue cases are confirmed in the laboratory by IgM antibody-based method (MAC ELISA). A list of all confirmed dengue cases from January 2012 to December 2019 reported in Bhopal city was obtained from the District Malaria Office, Bhopal. This data contained date wise confirmed dengue cases along with their sex, age and address reported from government as well as private hospitals. The locations of the reported cases were geotagged using Google Earth [48]. The dengue cases based on the date of reporting were entered in the SPSS software, and weekly and monthly dengue cases were aggregated for statistical and spatial analysis. The week wise dengue incidence data and their ward wise distribution has been deposited in Excel format in figshare repository as an open access link (https://figshare.com/articles/dataset/An_assessment_of_remotely_sensed_environmental_variables_on_Dengue_epidemiology_in_Central_India/20765668). The study was approved by the Institutional Ethics Committee of ICMR-National Institute for Research in Environmental Health, Bhopal (NIREH/BPL/IEC/2018-19/3130 dated 18-03-2019).

#### Collection of meteorological data

Daily average values of rainfall (mm), maximum, minimum and mean temperature (°C), and RH (%), all measured at 2 metres from ground level, were collected from 1^st^ January 2012 to 31^st^ December 2019. The parameters were extracted from the National Aeronautics and Space Administration (NASA) Langley Research Center Prediction of Worldwide Energy Resource Project [49], which is based on Modern-Era Retrospective analysis for Research and Applications version 2 (MERRA-2) dataset merged with Goddard Earth Observing System Model (GEOS) version 5.12.4. Absolute humidity (AH, g/m^3^), was estimated using dry bulb temperature and RH at standard atmospheric pressure [50] using the following equation:

$$AH=1000\times (6.11\times 10^{T_{1}}\times 100)/(T_{C}+273.16)\times 461.5$$ (1)

Where, Tc is the dry bulb temperature (in our studies, Tc is the daily mean temperature), and

$$T_{1}=7.5\times T_{d}/(237.7+T_{d})$$

Where, Td is the dew point temperature. Td is approximated from the equation below, based on dry bulb temperature and RH:

$$T_{d}=(‐430.22+237.7\times \ln(E))/(‐\ln(E)+19.08),\;\mathrm{where},$$

$$E=RH\times E_{S}/100$$

$$E_{s}=6.11\times 10^{T_{2}}$$

$$T_{2}=7.5\times T_{c}/(237.7+T_{c}),$$

Daily values of weather variables were converted to weekly values as simple arithmetic sums for rainfall and means for other parameters such as temperature, humidity etc., to assess the potential effect of seasonal variation on dengue dynamics.

#### Extraction and processing of remote sensing data

The multi-sensor satellite images were extracted through Google Earth Engine [48] and ArcGIS 10.2 software [51] to assess the change in land use/land cover (LULC) and vegetation index. Landsat-8 Operational Land Imagers (OLI) and Thermal Infra-Red Sensors (TIRS) images (S1 Table) were extracted for preparing LULC maps with nine LULC classes for two time periods (2014 and 2020) using the spectral signatures of multi-bands through the visual image interpretation approach. The accuracy of delineated LULC classes was assessed with the help of high-resolution true-colour Google Earth images and the LULC change transition matrix was prepared.

In addition to LULC, vegetation and built-up indices were prepared to assess the correlation between prominent land use variables and incident dengue cases. The Normalized Difference Vegetation Index (NDVI) is a numerical quantity derived from reflectance measured in the Near-Infrared (NIR) and Red spectral bands. NDVI provides consistent spatial and temporal comparisons of vegetation canopy greenness, a composite property of leaf area, chlorophyll and canopy structure [52,53]. The NDVI can be determined using the following equation.


NDVI=(NIR–Red)/(NIR+Red) (2)

The Moderate Resolution Imaging Spectroradiometer (MODIS) NDVI data of the Terra satellite (MOD13Q1) products were used to estimate the monthly average NDVI value for Bhopal city in GEE platform for 96 months (January 2012 to December 2019) and was aggregated by ward using ArcGIS spatial statistics tool. Cross-correlations between the monthly averaged NDVI values and monthly cases were evaluated using Pearson’s product-moment correlation coefficient (*r*^2^).

The LULC data downloaded from Copernicus Global Land Cover Service (CGLS) [54] was used to summarize ward wise annual built-up area from 2015 to 2019 using the ArcGIS 10.2 Spatial Analyst extension [51]. Furthermore, built-up areas (values ≥1) were extracted, and ward wise built-up areas were computed using zonal statistics in the ArcGIS 10.2 software. The ward wise built-up density was calculated by dividing the built-up area by the total area of each ward. A stepwise backward multiple linear regression was used to evaluate the influence of these landscape variables (ward wise maximum, minimum, and average NDVI values along with built-up density) on dengue incidences.

#### Statistical analyses

Basic demographic characteristics such as numbers, median age, gender and age-group of dengue cases reported in Bhopal city during the study period (2012–2019) were analyzed year-wise. Continuous variables were summarized as mean and standard deviation when normally distributed, and median with inter-quartile range when non-normally distributed. Categorical variables were summarized as counts and proportions. For all analyses, p-value <0.05 was considered statistically significant. Time series of weekly dengue case count were plotted along with weekly average values of mean minimum and maximum temperature, mean RH, and total precipitation.

The annual average rate of change in the maximum, minimum and mean temperature over the study period in Bhopal city were estimated by the Mann-Kendall non-parametric test and Sen’s slope estimate [55]. In this estimate, if “*n*” is nine or less, the test statistic S is applied. The absolute value of S is compared to the probabilities of the Mann-Kendall non-parametric test to study if there is a monotonic trend or not at the significance level of α. A positive (or negative) value of S indicates an upward (or downward) trend. The Sen’s slope estimate, Q is used to estimate the actual slope of the linear trend (change per year). An α value of 0.1 is considered to be statistically significant [55].

#### Spatial analysis

Collected epidemiological and geographical data were converted into spatial layers and aggregated to the ward boundaries of Bhopal city using ArcGIS 10.2 software [51]. The ward wise cases from 2012 to 2019 were analyzed and normalized with the wards’ total population (as per the 2011 census). The normalized cases per 10,000 inhabitants were used to obtain the spatial-temporal distribution of dengue cases in Bhopal city.

Spatial autocorrelation analysis was performed for case locations and ward wise case distribution using the spatial statistics tool in ArcGIS 10.2 software [51]. The optimized hotspot analysis through Getis-Ord Gi*statistic was applied to date-wise locations of dengue cases by creating fishnet polygon mesh (cell size = 150 m) for aggregating the incidents. This spatial statistic works by looking at values of input variables for each case/ward location within the context of the neighbouring cases/wards. The local sum for each fishnet polygon and its neighbors is compared proportionally to the sum of all neighbouring cases/wards. The corresponding Z-score is calculated using Getis-Ord Gi*statistic. To have a significant hotspot of dengue incidence, it should have a location with high case density and adjacent areas with high dengue cases. The analysis found 506 statistically significant locations based on false discovery rate (FDR) correction for multiple testing and spatial dependence of dengue incidence. The locations/wards with high values of positive Z-scores (>1.96) are referred to as ‘hotspots’, and negative Z-scores are termed as ‘coldspots’. If there are no hot or coldspots, the cases/wards would have a random spatial distribution.

#### Estimation of lag effects

To investigate the delayed impact of meteorological variables on the incidence of dengue cases, we employed two distinct approaches to assess this relationship. First, the association between weekly dengue incidence and meteorological variables at different time lags (0–30 weeks) was assessed using Pearson’s cross-correlation analysis. Lag represents the time gap between exposure and clinical outcomes. The second part of the analysis was carried out using the distributed lag non-linear model (DLNM) of the “*dlnm*” package [56,57] available in the R software, v3.3.8 [58]. This model can examine non-linear relationships and addresses multi-collinearity issues by applying spline smoothing techniques or polynomial functions. It can handle lag effects and non-linear relationships simultaneously using a bi-dimensional function. The delayed effect is important in dengue transmission and climate fluctuations related to the duration of the mosquito life cycle and virus propagation [59]. Many studies have used this approach to evaluate non-linear relationships between climatic factors and mosquito-borne diseases [45,50,59,60].

DLNM combined with quasi-Poisson regression was used to calculate relative risk (RR) by estimating the effect of independent variables on the dependent variable. This model is represented for time series data by the following equation (Eq 3):

$$Y_{t}=\mathrm{Quasi}‐\mathrm{Poisson}\;(\mu_{t}),\;t=1,2,3,\ldots \ldots \ldots ,n$$

$$Log\;(\mu_{t})=\alpha +\sum_{l=1}^{L}\beta 1(T_{t,l})+\sum_{l=1}^{L}\beta 2(R_{t,l})+log{(N}_{t})+s(t,\lambda )+Year+\varepsilon_{t}$$ (3)

Where, *t* is the week of the observation, *Y*_*t*_ denotes the observed dengue counts in week *t*, *log (μ*_*t*_*)* represents the logarithm of expected dengue cases in week *t*, α is the model intercept; *T*_*t*,*l*_, and *R*_*t*,*l*_, are the matrices obtained by applying the DLNM to; *β*_*1*_, and *β*_*2*_ are the vectors of coefficients for *T*_*t*,*l*_ and *R*_*t*,*l*_; *Ɩ* is the lag in weeks; *L* is the maximum lag (in our case it is 25 weeks); *N*_*t*_ is an offset to control for population using a linear function of time based on the 2001 and 2011 censuses population of Bhopal; *s (t*, *λ)* is the natural cubic spline smoothing function of the calendar week and *ε*_*t*_ are the residuals added at specific lags to correct for partial autocorrelation. The mean values of each climatic variable (S2 Table) were used as reference to calculate RR.

Identification and removal of seasonality from time-series data will improve the modeling performance and will also result in a clean relationship between the input and output variables. Therefore, all climatic variables were tested for seasonality before using the DLNM modeling approach. This considerably decreases the collinearity problem while maintaining the model’s interpretability. The MSTL (Multiple Seasonal-Trend decomposition using Loess) time series seasonal adjustment method was used using the “*mstl*” function in R [61], and seasonality adjusted data was extracted using the "*seasadj*" function included in the "*forecast*" package in R [62].

Similarly, the delayed effect of monthly average values of NDVI to dengue incidences was also assessed using the DLNM method.

The numerical data used in for figures are included in S1 Data.

## Results

### Descriptive epidemiology of dengue in Bhopal

**Fig 2:**
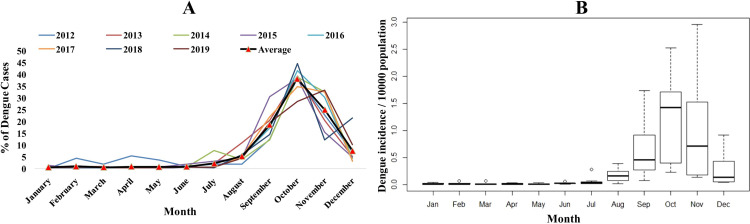
Month wise distribution of dengue cases in Bhopal city (2012–2019). A) Month wise percentage contribution of dengue cases. B) Month wise box plot distribution of dengue case incidence per 10,000 inhabitants (The box comprises 50% of the distribution, the line within the box is the median value, borderlines are the first and the third quartile, and the small circles are the outliers. February, March, June, and July had outliers).

A total of 5,333 dengue cases were notified in Bhopal city during 2012–2019 (Table 1). A gradual increase in the dengue cases was observed over the study period. The case incidence varied from 0.59 cases/10,000 inhabitants (95% CI: 0.49–0.71) in 2012 to 9.11 cases/10,000 inhabitants (95% CI: 8.69–9.55) in 2019. The case incidence in Bhopal city was higher than the average of the entire state of Madhya Pradesh and India during the study period (S1 Fig). From 2012–2019, Bhopal witnessed a 14.5-fold increase in annual dengue incidence, while it was a 2.13-fold increase at the national level. More than 50% of the cases were males irrespective of the age groups (Table 1). The median age for the notified cases was 24 years (IQR: 14.5–32 years) with a male to female ratio of 1.8:1. The majority of the cases (60.7%) belonged to 18–45 years age group, and was followed by 9–17 years age group (17.5%). Dengue cases increased sharply during September to November, contributing to almost 82% of the yearly case load. The cases peaked in October, which accounts for 38% of the yearly cases (Fig 2A and 2B).

**Table 1:** Demographic characteristics of notified dengue cases in Bhopal city (2012–2019).

Characteristics	2012	2013	2014	2015	2016	2017	2018	2019	Total
**Number of cases**	114	158	706	232	661	1094	615	1753	5333
**Case Density/ 10,000 populations (95%CI)**	0.59 (0.49–0.71)	0.82 (0.70–0.96)	3.67 (3.41–3.95)	1.21 (1.06–1.37)	3.44 (3.18–3.71)	5.69 (5.36–6.03)	3.20 (2.95–3.46)	9.11 (8.69–9.55)	27.72 (26.99–28.47)
**Median Age, years (IQR)**	23 (11–33)	22 (18–33)	24 (16–35)	23 (17–34)	23 (17–35)	25 (18–39)	24 (16–33)	24 (18–35)	24 (14.5–32)
**Sex, number (%)**									
**Male**	71 (62.3)	112 (70.9)	441 (62.5)	164 (70.7)	429 (64.9)	712 (65.1)	381 (62.0)	1128 (64.3)	3438 (64.5)
**Female**	39 (34.2)	46 (29.1)	265 (37.5)	68 (29.3)	232 (35.1)	379 (34.6)	210 (34.1)	593 (33.8)	1832 (34.4)
**Unspecified**	4 (3.5)	0 (0.0)	0 (0.0)	0 (0.0)	0 (0.0)	3 (0.3)	24 (3.9)	32 (1.8)	63 (1.2)
**Age group (%)**									
**<5 Years**	10 (8.8)	5 (3.2)	28 (4.0)	10 (4.3)	14 (2.1)	31 (2.8)	25 (4.1)	47 (2.7)	170 (3.2)
**5–8 Years**	8 (7.0)	10 (6.3)	32 (4.5)	11 (4.7)	22 (3.3)	22 (2.0)	38 (6.2)	48 (2.7)	191 (3.6)
**9–17 Years**	22 (19.3)	22 (13.9)	149 (21.1)	40 (17.2)	130 (19.7)	183 (16.7)	95 (15.4)	291 (16.6)	932 (17.5)
**18–45 Years**	52 (45.6)	102 (64.6)	409 (57.9)	144 (62.1)	411 (62.1)	669 (61.2)	372 (60.5)	1079 (61.6)	3238 (60.7)
**> 45 Years**	15 (13.2)	19 (12.0)	81 (11.5)	27 (11.6)	83 (12.6)	179 (16.4)	63 (10.2)	225 (12.8)	692 (13.0)
**Unspecified**	7 (6.1)	0 (0.0)	7 (1.0)	0 (0.0)	1 (0.2)	10 (0.9)	22 (3.6)	63 (3.6)	110 (2.1)

### Ward wise distribution of dengue cases and dengue hotspots

**Fig 3:**
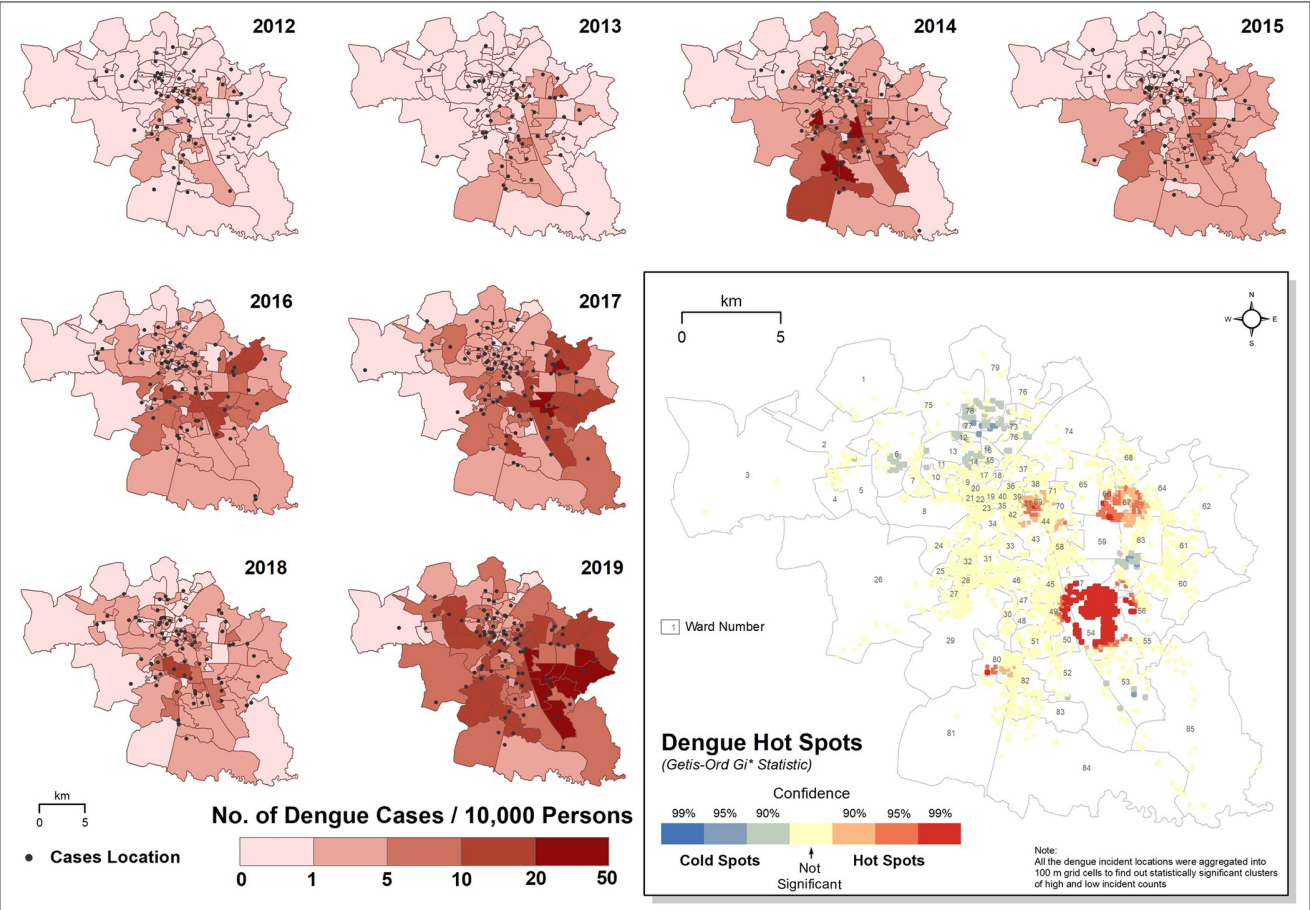
Ward wise spatial distribution of dengue cases (2012–2019) indicating significant hotspots and coldspots in Bhopal city. The hotspots are shown in the map as shades of red to highlight the geographic areas where the clustering of dengue cases occurred. Similarly, coldspots with negative Z-scores are shown in blue to indicate areas where fewer-than-expected dengue cases were observed. (Source of shapefile: http://projects.datameet.org/Municipal_Spatial_Data/).

Bhopal city witnessed a gradual expansion of dengue cases over the years (S1 Fig). Of the 85 wards, only 5 wards had >2.0 dengue cases/10,000 inhabitants in 2012, while in 2014 and 2019, 38 and 75 wards had >2.0 cases/10,000 inhabitants, respectively (S2 Fig), suggesting most of the city had sustained transmission of dengue. Spatial distribution showed the wards located in the southern and eastern parts of the city to be the significant dengue hotspots (Fig 3).

### Effect of meteorological variables on dengue incidence

**Fig 4:**
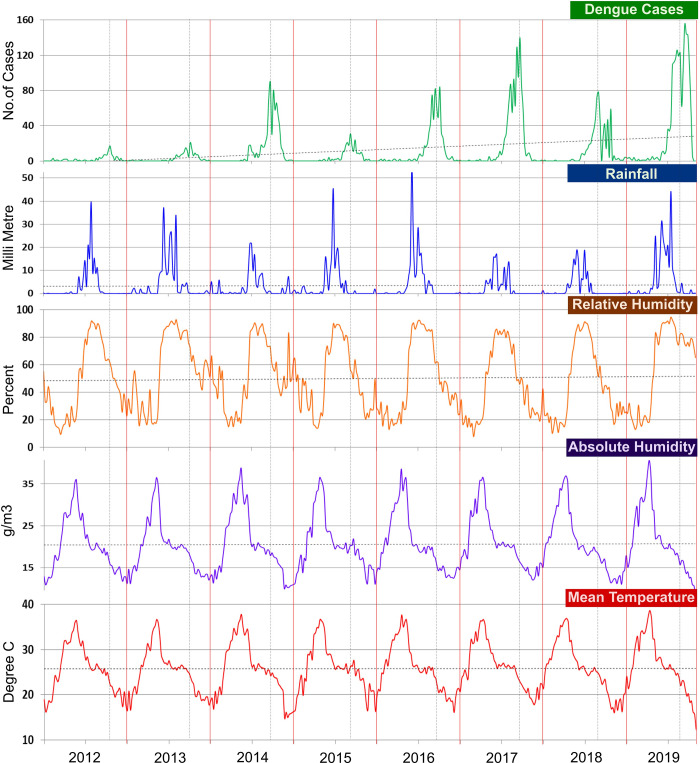
Time–series plots of weekly dengue cases, weekly cumulative rainfall, relative humidity, absolute humidity and mean temperature (2012–2019).

The weekly dengue cases, mean temperature, rainfall, RH and estimated AH from 1^st^ January 2012 to 31^st^ December 2019 in Bhopal city are presented in Fig 4. On an average, 13 cases per week and 664 cases per year were reported, with maximum cases occurring during weeks 38–45 (mid-September to mid-November) of the year. Descriptive statistics of all meteorological variables are shown in S2 Table. Even though the yearly peak of dengue cases varied slightly from year to year, most cases tended to occur following the monsoon season (Fig 4).

The weekly average of meteorological variables such as rainfall, humidity, diurnal temperature range, mean temperature, and AH exhibited significant Pearson cross-correlation correlations with weakly dengue cases at different time lags (S3 Fig). A significant correlation between dengue and rainfall (*r*^2^ = 0.510), humidity (*r*^2^ = 0.554), diurnal temperature range (*r*^2^ = 0.601) and temperature (*r*^2^ = 0.599) were observed at 12, 8, 9 and >20 weeks lag respectively (S3 Fig).

**Fig 5:**
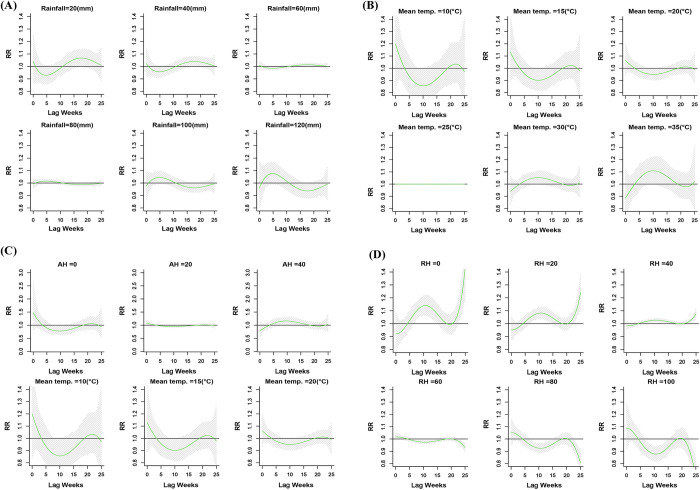
Association of dengue incidence with different ranges of meteorological variables. A) RR by lag weeks at different rainfall (mm) levels. B) RR by lag weeks at different mean temperature (°C). C) RR by lag weeks at different AH (g/m^3^). D) RR by lag weeks at different RH (%). The solid green line is the estimated linear curve, with shaded region indicating its 95% confidence interval.

As no effect of seasonality was observed on the climatic variables based on the seasonality decomposition analysis (S4 Fig), the specific correlation of the climatic variables to dengue incidence was assessed by DLNM using the original data set. The delayed effect/lagged relationship between weekly meteorological variables (rainfall, mean temperature, AH) and dengue cases, analyzed using DLNM combined with quasi Poisson regression analysis, revealed a non-linear relationship (S5 Fig). When compared to the drier weeks (weekly rainfall <50mm), the wettest weeks (weekly rainfall >100mm) have a shorter lag period of 5 weeks with a RR of 1.08 (95% CI [0.99–1.17]) (Fig 5A). The association between mean temperature and dengue cases with a 25 week lag is shown in S5B Fig. The RR was 1.11 (95% CI [1.01–1.22]) at 35°C mean temperature with a 5–15 week-lag (peak at 10 weeks) compared to the reference value (25.9°C) (Fig 5B). Similarly, risk of dengue cases increases with increasing AH (S5C Fig). The RR of dengue cases was 1.42 (95% CI [1.07–1.88]), 1.73 (95% CI [1.11–2.71]) and 2.11 (95% CI [1.15–3.89]) for AH 60, 80 and 100 g/m^3^ respectively with a 5–15 week lag (peaked at 10 weeks) (Fig 5C). The RH appears to follow the pattern of rainfall on RR of weekly dengue incidence; however, the DLNM model does not show any significant association (Figs 5D and S5D).

### Effect of urbanization on dengue incidence

The LULC change between 2014 and 2020 (Table 2) for Bhopal city (S6 Fig) revealed a 9.1% increase in the built-up classes and a 3% reduction in the agricultural land use. The crop land surrounding the city decreased considerably, and since 2013, greater than half of the agricultural land use (~765 ha) was converted to open scrub and built-up with vegetation. To understand the dominance of impervious cover, a yearly built-up area map was prepared for Bhopal city from 2015–2019, and built-up density was calculated for each ward. Since both vegetation cover and built-up density can be considered as a proxy for urbanization, we further computed the ward wise average minimum and maximum NDVI values along with built-up and population density. To better understand the role of these remotely sensed landscape variables on dengue incidence, a backward stepwise regression was applied. Minimum NDVI was the significant landscape variable influencing dengue incidence in Bhopal city (model *r*^2^ = 0.035, F statistics = 16.293, *p =* 0.0001) (Table 3).

**Table 2:** LULC Matrix for changes in hectares (ha) in different land cover class (2014 to 2020).

Land use area-2020												
**Land use area—2014**	**Classes**	Agriculture	Dense scrubs	Open scrubs	Dense Built-up	Built-up with vegetation	Sparse Built-up	Structures/Misc.	Water Body	Fallow/Barren land	**Land use area (ha) 2014**	Change in Land use (ha) (2014–2020)
	Agriculture	45230.1	1.0	339.2	156.3	425.0	357.6	123.9			**46633.1**	-1401.5
	Dense scrubs	1.0	2077.9	11.1	0.1	1.1			3.9		**2095.1**	35.0
	Open scrubs	0.6	33.6	10372.5	94.7	77.5	45.9	94.6	14.9	17.9	**10752.2**	-9.1
	Dense Built-up				5939.1	0.1		1.3			**5940.5**	407.5
	Built-up with vegetation				81.9	5298.0					**5379.9**	421.9
	Sparse Built-up				75.9		2585.9				**2661.7**	358.5
	Structures/Misc.							1452.2			**1452.2**	219.8
	Water Body		17.7	15.7					5953.5	4.6	**5991.5**	-16.2
	Fallow/Barren land			4.6			30.9		2.9	2021.2	**2059.7**	-16.0
	**Land use area (ha) 2020**	**45231.6**	**2130.1**	**10743.1**	**6348.0**	**5801.8**	**3020.3**	**1672.0**	**5975.2**	**2043.7**	**82965.9**	

**Table 3:** Hierarchical multiple regression analysis with backward elimination of landscape variables on dengue incidence in Bhopal city.

Covariates	B	SE	Adjusted *r*^2^	t	*p*
Model 1					
Intercept	2.304	1.413	0.037	1.631	0.104
Built-up Density	-1.273	0.742		-1.716	0.087
NDVI_Average	12.257	14.378		0.852	0.394
NDVI_Maximum	-10.352	8.227		-1.258	0.209
NDVI_Minimum	16.696	6.68		2.499	0.013
Model 2					
Intercept	2.513	1.391	0.038	1.806	0.072
Built-up Density	-1.25	0.741		-1.687	0.092
NDVI_Maximum	-3.836	3.043		-1.261	0.208
NDVI_Minimum	20.093	5.36		3.748	0
Model 3			0.037		
Intercept	1.482	1.127		1.316	0.189
Built-up Density	-0.917	0.693		-1.324	0.186
NDVI_Minimum	15.906	4.211		3.778	0
Model 3					
Intercept	0.626	0.923	0.035	0.678	0.498
NDVI_Minimum	16.794	4.161		4.037	0
Dependent variable: Dengue case density per 10000 inhabitants					
F-Statistics: 16.293					
*p*-value of F-statistics: 0.0001					

B = Coefficient, SE = Standard error

A slight increase in the monthly average NDVI and a corresponding increase in dengue cases were observed (S7A Fig). The cross-correlation of monthly average NDVI with dengue cases over the period was significantly positive (*r*^2^ = 0.303) in the same month (0 month lag period) (S3 Table). The strength of association slightly increased (*r*^2^ = 0.339) after a lag period of one month. Dengue incidence with RR of 1.42 (95% CI [0.99–1.84]) was observed at >4 months lag at average monthly NDVI value of 0.2 using the DLNM approach (S7B Fig).

## Discussion

Understanding the epidemiology of dengue in the context of environmental factors is important for its better management and control. In India, except for New Delhi [63], the role of climatic and landscape variables on dengue incidence and transmission at the city level has been poorly studied. We examined the relationship of various meteorological and landscape variables with dengue incidence in the city of Bhopal, Central India. Our study found increasing mean temperature (30°-35°C) with 5–15 week-lag, low rainfall (20-40mm) with a longer lag (15–20 weeks), high rainfall (above 100mm) with a shorter lag (5–10 weeks) and high AH (above 60 g/m^3^) with a lag of 5–15 weeks to be the most significant meteorological variables associated with an increased dengue incidence in Bhopal city.

Temperature is a key climatic factor that affects both the life cycle of vector mosquitoes as well as the pathogen it harbors. Temperature influences the length of the gonotrophic cycle, larval development and the growth rate of mosquitoes [17]. An increase in temperature is associated with an increased dengue incidence due to faster viral replication rate, shortened EIP, and increased mosquito biting rate, leading to higher vectorial capacity and disease transmission [23,64,65]. DLNM shows a positive association of dengue with 30°-35°C at a lag of 5–15 weeks. This mean temperature range (30°-35°C) is usually maintained from mid-April to end of June in Bhopal (dry season). The desiccation resistant ability of *Aedes* eggs [66], and trans-ovarian transmission capability of dengue virus through desiccated eggs [67] help them sustain the dry season which is followed by rainy season from mid-June allowing *Aedes* eggs to hatch, expand *Aedes* population and establish the dengue transmission cycle. In line with our findings, an association of elevated mean temperature with a long lagged dengue incidence (3–5 months) was also observed in Singapore [18,50,66,68], Nepal [69], and Thailand [29]. Similarly Lowe et al. also observed drought conditions positively associated with dengue infection at a longer lag up to 5 months in Barbados [70]. Interestingly, heat waves in Singapore were also associated with a reduction in dengue infections; a 1°C increase from 31 to 32°C in maximum temperature was associated with a 13.1% decrease in dengue incidence over 6 weeks [60]. In India, the risk of dengue transmission increases with an increase in mean temperature above 24°C, and the greatest transmission risk was reported to be at 30°C with a lag of 0–3 weeks [45]. India has different climatic zones and predicting the risk of dengue based on climatic variables at the national level may not correlate well in the country’s diverse geographical settings. The average long-term temperature of major cities of India varies considerably due to its physiographic settings, for instance, the annual mean maximum temperature ranges 19°-41°C, 26°-32°C and 28°-37°C for Delhi, Bengaluru and Chennai cities respectively [71,72]. Clearly, the lag effect of temperature in different geographical settings in India needs to be ascertained to predict and plan future dengue control strategies.

Rainfall provides breeding habitats for mosquitoes, and *Ae*. *aegypti* density is an important variable and a predictor of dengue incidence in urban settings. Even though continuous heavy rainfall can washout breeding sites and negatively impact *Ae*. *aegypti* density [23,73,74], the extent of its effect is dependent on the intensity of rainfall, frequency of flush out events, size of the water holding containers, and larval age [75–77]. In Bhopal, rainfall and humidity increases from mid-June to September, and the mean temperature also rises above 30°C from April to June, and is accompanied by a rise in dengue cases; the surge in dengue cases is synchronized and correlated with delayed rainfall and temperature. Historically, in Bhopal, the number of dengue cases is low during the winter and dry period (January to May), and the peak is observed towards the end of the monsoon season (October). Many studies have reported a non-linear relationship between rainfall and dengue incidence. Increase in rainfall has shown to be positively associated with dengue, but with varied lag periods; in Malaysia, Sri Lanka and Brazil, the lag period is 4, 15–20 and 12 weeks respectively [78–80]. On a nationwide scale in India, the risk of dengue was highest at 60mm rainfall with a 12 week lag [45]. Dengue incidence in Bhopal is positively associated with high rainfall (100-120mm) and a shorter (5–10 weeks) lag period. Again, these findings underscore the importance of studying the meteorological variables in different climatic zones for efficient modeling of dengue transmission.

Most of the studies investigating the effect of environmental variables on dengue incidence have focused on temperature, rainfall and RH, while the effect of AH on dengue incidence has not been well described. We found the highest risk (RR: 1.42 to 2.11) of dengue incidence at a lag of 5–15 weeks above 60 g/m^3^ AH in Bhopal. In Singapore, a similar effect of AH, rather than RH, on dengue incidence was observed [50], and it was found to be a better predictor of dengue incidence with respect to other climatic variables. In Guangzhou province, China, higher RH was negatively associated with dengue incidence [59]. Thus, along with the mean temperature (which provides a suitable environment for virus replication within vector mosquitoes), AH (which amplifies the virus transmission potential) may provide a more stable and better predictor for modeling dengue incidence, and must be considered together with other contributing factors for an effective dengue control policy recommendation.

In addition to climatic variables, urbanization and anthropogenic influences are also considered as the key drivers of dengue expansion [81]. *Ae*. *aegypti*, the highly dominant, adaptable and domesticated dengue vector, is highly anthropophilic, and prefers to live alongside humans, hence, urbanization and population growth provide ideal opportunities for the breeding and spread of *Ae*. *aegypti*. A positive temporal association of monthly average NDVI and dengue incidence at a lag of one month was observed using Pearson’s cross-correlation. More specifically a higher risk of dengue was observed at a lower monthly average NDVI value with extended lag periods in comparison to higher monthly average NDVI values (S7B and S7C Fig). Similarly, the multiple regression model also suggests minimum NDVI as a significant risk factor for dengue incidence in the wards of Bhopal (Table 3). A higher dengue incidence rate was also found to be associated with low vegetation cover in São Paulo, Southeastern Brazil [82]. The maximum average values of NDVI for Bhopal city was mostly below 0.5 (S4 Table) which suggests sparse vegetation is connected with the built-up areas [83]. This alternatively explains the association of most of the dengue incidences with the high built-up areas in Bhopal. LULC classification of Bhopal shows an increase of 9.1% in built-up areas in 2020 when compared to 2014 (Table 2), and is in agreement with an earlier report [84]. Agent-based modeling shows Bhopal has grown rapidly from 1973 to 2014, with an intensification of urbanization from the city’s center to the periphery, and a predicted increase in built-up areas by 240–245% between 2014 and 2022 [84]. In Bhopal, agricultural land has decreased by 45%, while the built-up areas have increased by ~260% between 1972 and 2016 pointing to rapid urbanization [85]. An increase in the built-up area was also evident from the extracted built-up area map for the period 2015–2019 (S8 Fig). In Bhopal, apart from the core urban areas, most of the urban area expansion occurred in the southern part of the city since 2010 [86]. A statistically significant dengue hotspot towards the southern part of the city (Fig 3) also signifies the influence of increasing urbanization on dengue incidences in Bhopal city. The slightly negative correlation (*r*^2^ = - 0.093) between the association of built-up area with dengue incidence might be because of the relatively longer time period required to capture the changes in the built-up areas. Future studies should explore decadal trends to assess the spatio-temporal association of increase in built-up areas to dengue incidence. Furthermore, other socio-demographic factors, such as the increase in population density in comparison to built-up areas are also important to assess the role of urbanization on dengue incidence. From 2000–2015, Bhopal’s population and built-up areas increased by 41% and 35% respectively [86]; this increase in population density might facilitate increased human-mosquito contact and could increase dengue incidence. Rapid urbanization has resulted in intense population growth (~200%) in Bhopal during the last 3 decades [85], and could be an important contributing factor for the increase in the monthly and annual mean temperature. In Bhopal, during the study period (2012–2019), the overall annual maximum, minimum, and mean temperature have increased by 0.1°C (Test S = 10, α = 0.1), and is in line with earlier findings from Bhopal [87] and Mizoram [88]. The increase in built-up areas, along with the increase in monthly and annual mean temperature will provide suitable breeding habitats for *Ae*. *aegypti* to proliferate and transmit the dengue virus.

Even though the present study has helped understand the role of local environmental factors on dengue virus transmission in Bhopal, the study has limitations, especially related to the potential bias in the dengue incidence data. The data used in the study is only from clinically diagnosed cases from government and private hospitals, and may not represent the true burden of dengue in Bhopal. The majority of the dengue cases are mild or asymptomatic, and therefore it is very difficult to estimate the true annual burden of dengue in an endemic setting [89]. Furthermore, age, gender and socio-economic status are other important factors that influence the health-seeking behavior, and thus could bias the dengue case reporting. Correcting this reporting bias may lead to more accurate and robust estimation of the dengue burden [90]. The best approach to correct the bias is to derive a multiplication factor based on the socio-economic strata of the different wards in the study area [91]. However, in India, ward level economic datasets are not available in the public domain. Based on the 2011 census (latest available), we tried to extract indirect indicators for deriving ward level multiplication factors, but were not able to find a reliable indicator.

Overall, to the best of our knowledge, this is the first report of the effect of remotely sensed meteorological and landscape variables on dengue epidemiology at a city level in Central India. In addition to devising vector control strategies, our study methodology and findings can serve as a template for understanding and forecasting dengue transmission at micro-level in different dengue-endemic climatic zones of India.

## Supporting information

S1 Data Excel spreadsheet containing, in separate sheets, the underlying numerical data for Figs 2 and 4, Table 3, S1, S2 and S7 Figs and S2 Table. (XLSX)

S1 Fig Comparison of dengue incidence (2012–2019) in Bhopal city, Madhya Pradesh and India. (TIF)

S2 Fig Heat-map showing ward wise dengue case density in Bhopal city (2012–2019). (TIF)

S3 Fig Cross-correlation of dengue cases and weather variables at 0–30 weeks lag time. The dotted line stands for the highest *r*2—values with most significant correlation coefficient (p<0.0001).
(TIF)

S4 Fig **Seasonal-unadjusted versus seasonal-adjusted time series data for meteorological variables** (A) average temperature (°C), (B) maximum temperature (°C), (C) absolute humidity (g/m^3^) and (D) rainfall (mm).
(TIF)

S5 Fig **The three-dimensional plot showing the association between weekly** (A) rainfall (mm), (B) Mean temperature (°C), (C) absolute humidity (g/m^3^), (D) relative humidity (%) and relative risk (RR) of dengue at different week lags.
(TIF)

S6 Fig Changes in Land use Land cover patterns during 2014–2020 in Bhopal city. Land use land cover classes of the study area were visually interpreted by the authors and the map layer was generated using ArcGIS version 10.2 software as described in Methods.
(TIF)

S7 Fig (A) Time–series plot of monthly dengue cases with monthly average NDVI values (2012–2019), (B-C) Association of dengue incidence with different ranges of NDVI values.
(TIF)

S8 Fig Changes in built-up area in Bhopal city between 2015 and 2019 (Source of shapefile: http://projects.datameet.org/Municipal_Spatial_Data/). (TIF)

S1 Table Details of the satellite images used for land use land cover (LULC) classification of Bhopal city. (DOCX)

S2 Table Descriptive statistics of weekly values of weather variables from 2012 to 2019 in Bhopal city. (DOCX)

S3 Table Cross-correlation analysis between dengue cases and monthly NDVI at the different lag periods in Bhopal City. (DOCX)

S4 Table Changes in NDVI index in high endemic wards, low endemic wards and overall NDVI of Bhopal city from 2012–2019. (DOCX)
