# Supplementary figures and images for: An assessment of remotely sensed environmental variables on Dengue epidemiology in Central India
Source: PLoS Negl Trop Dis. 2022 Oct 17;16(10):e0010859. doi: 10.1371/journal.pntd.0010859 (PMC9612820; doi:10.1371/journal.pntd.0010859)

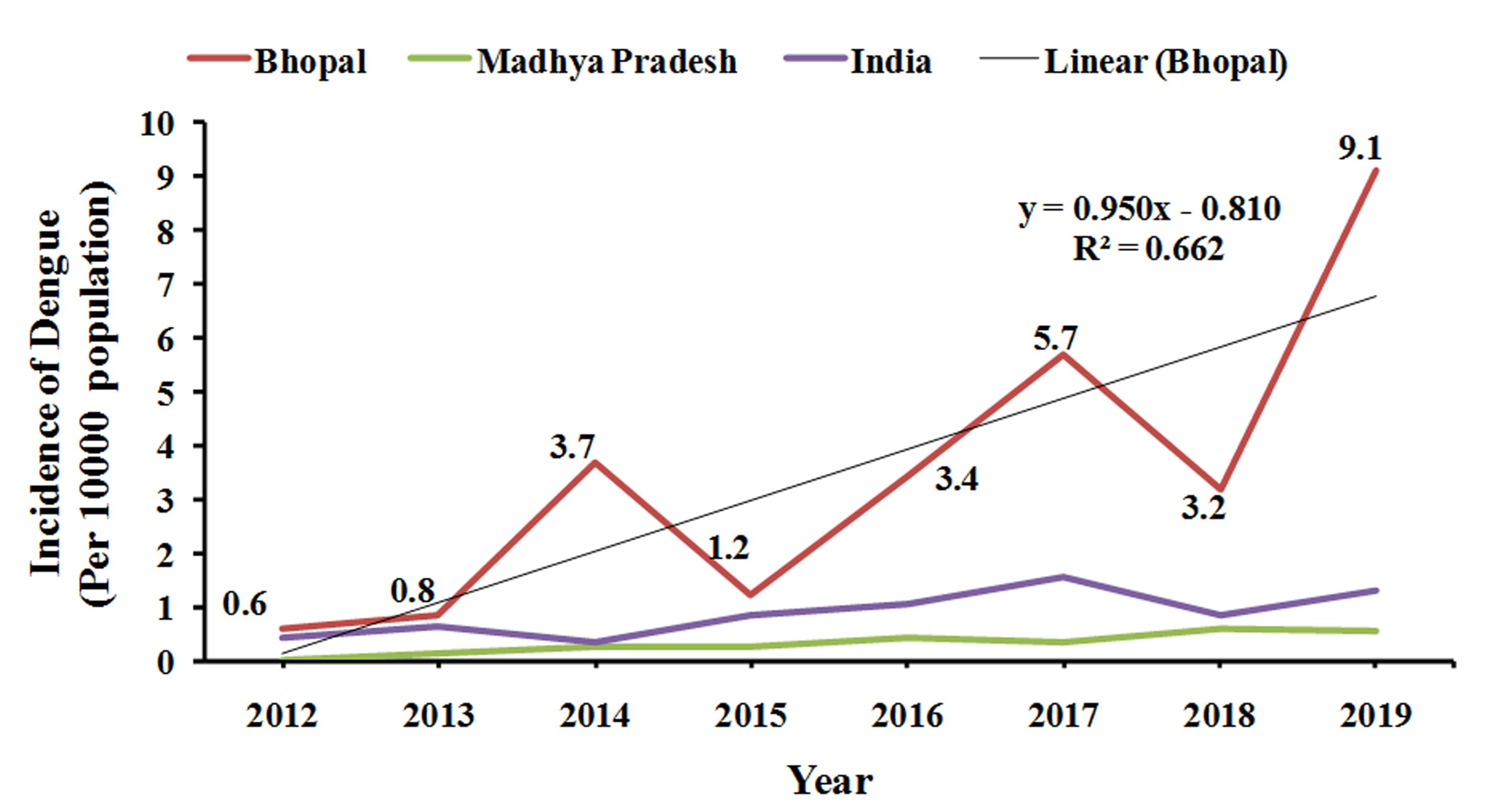

Supplement: S1 Fig — (TIF) [file pntd.0010859.s002.tif]

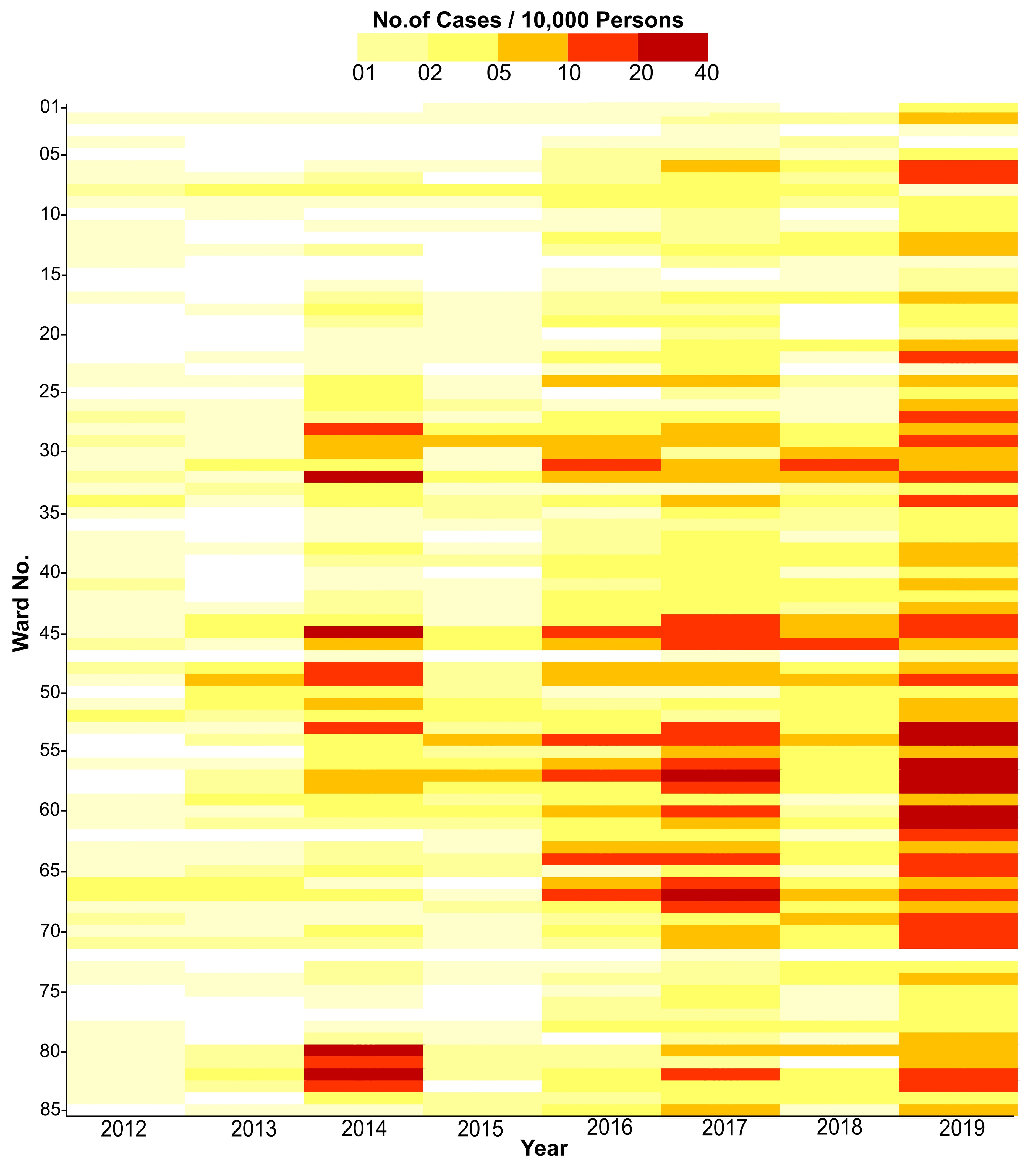

Supplement: S2 Fig — (TIF) [file pntd.0010859.s003.tif]

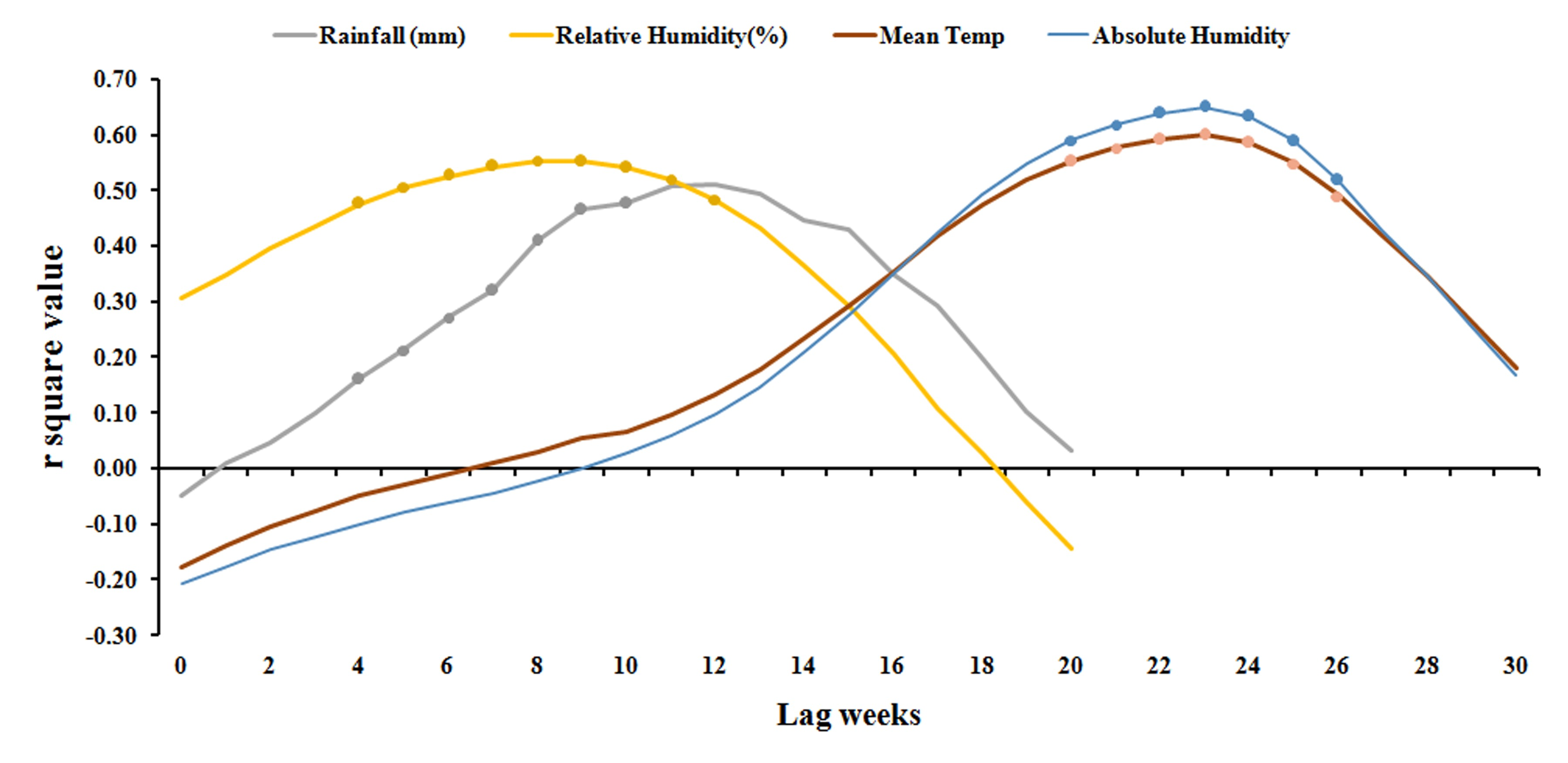

Supplement: S3 Fig — The dotted line stands for the highest r2—values with most significant correlation coefficient (p<0.0001). (TIF) [file pntd.0010859.s004.tif]

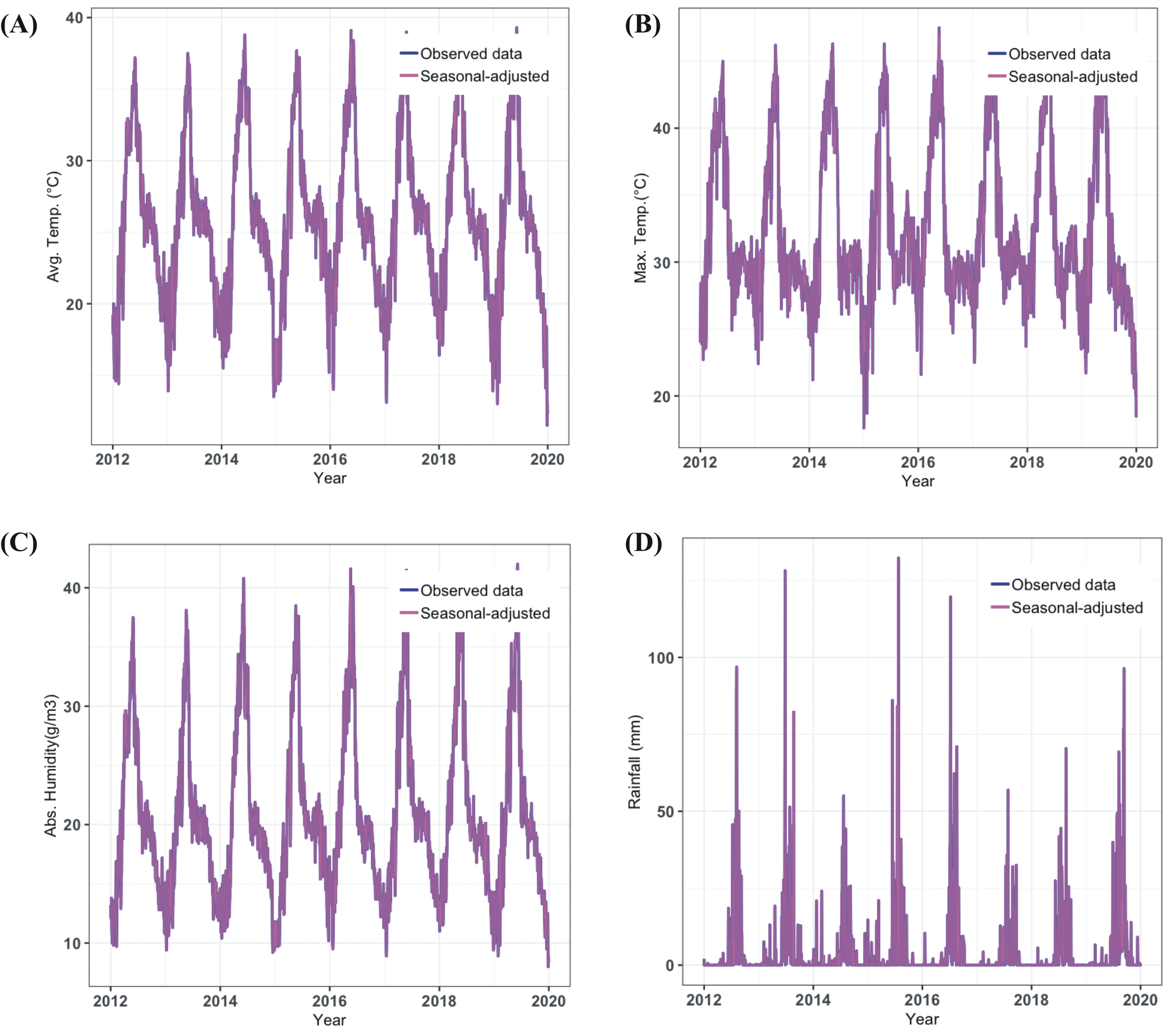

Supplement: S4 Fig — Seasonal-unadjusted versus seasonal-adjusted time series data for meteorological variables (A) average temperature (°C), (B) maximum temperature (°C), (C) absolute humidity (g/m3) and (D) rainfall (mm). (TIF) [file pntd.0010859.s005.tif]

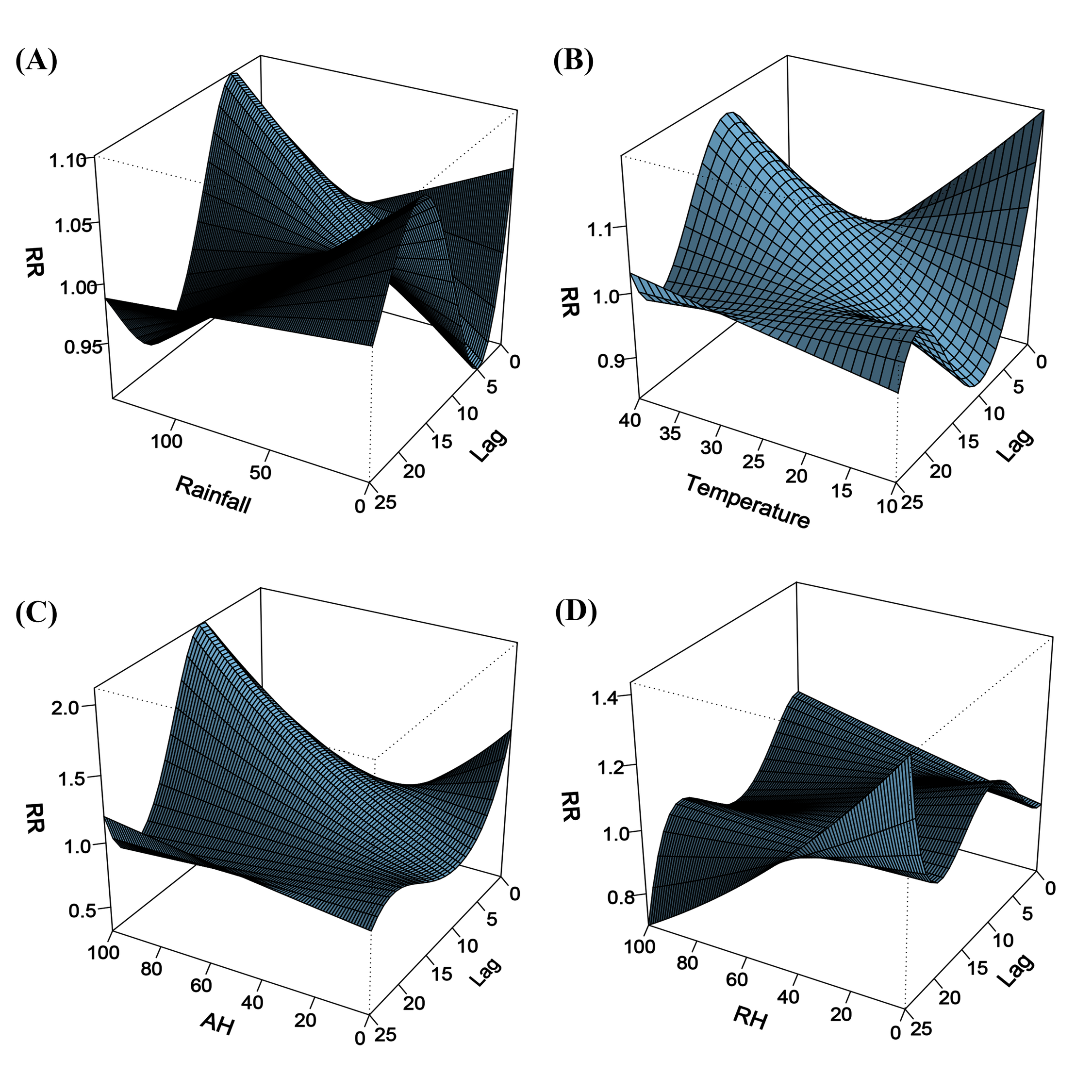

Supplement: S5 Fig — The three-dimensional plot showing the association between weekly (A) rainfall (mm), (B) Mean temperature (°C), (C) absolute humidity (g/m3), (D) relative humidity (%) and relative risk (RR) of dengue at different week lags. (TIF) [file pntd.0010859.s006.tif]

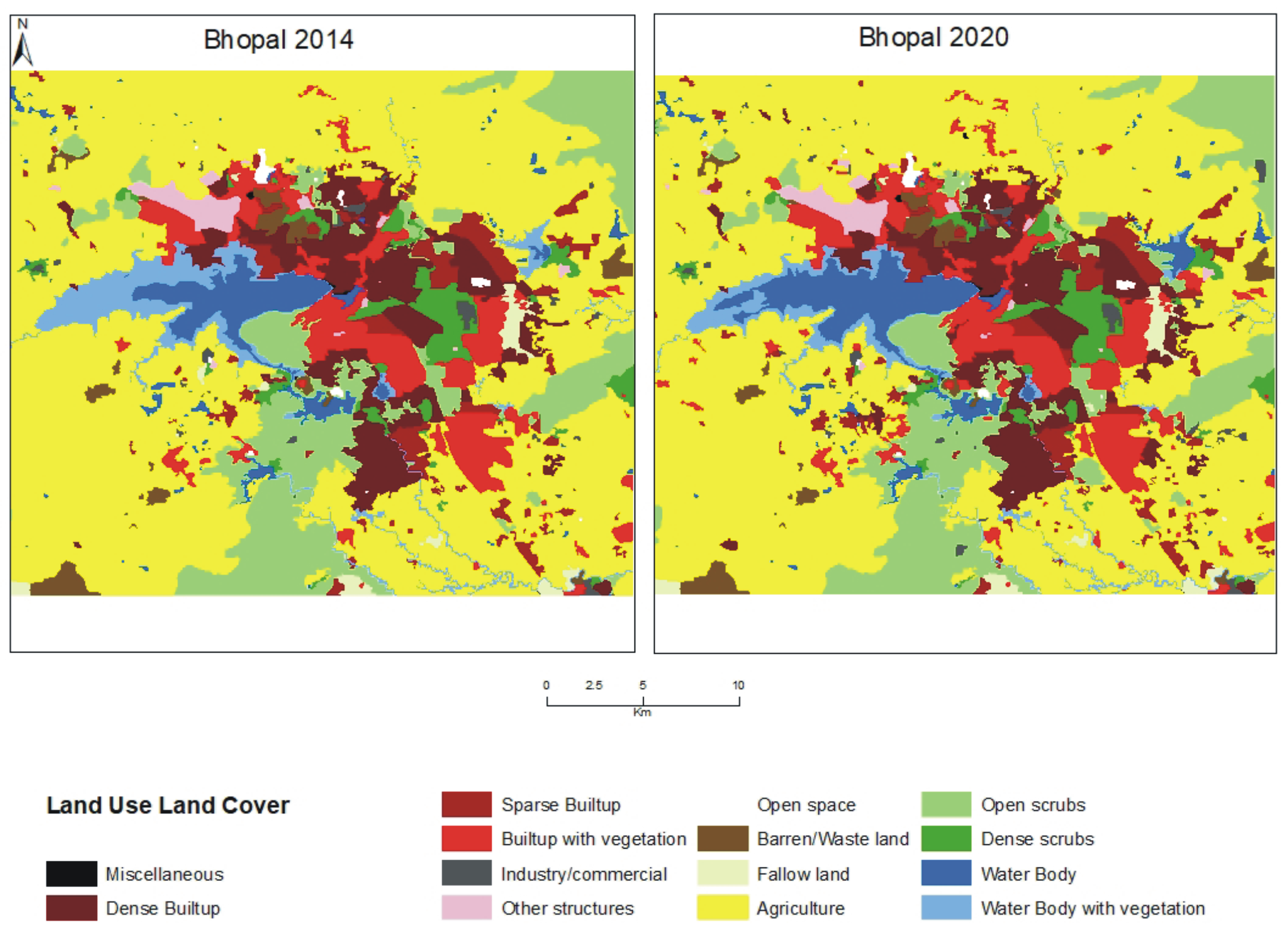

Supplement: S6 Fig — Land use land cover classes of the study area were visually interpreted by the authors and the map layer was generated using ArcGIS version 10.2 software as described in Methods. (TIF) [file pntd.0010859.s007.tif]

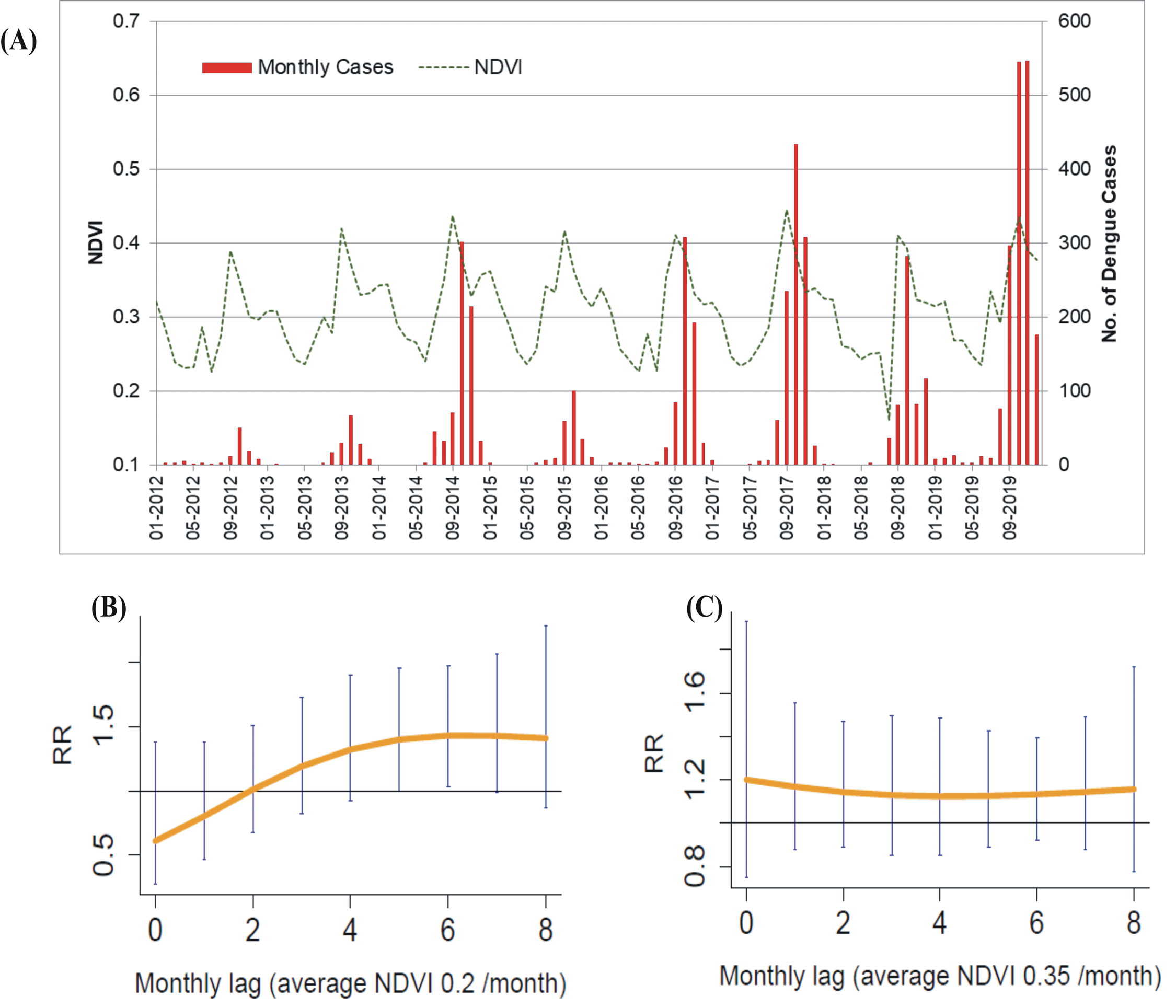

Supplement: S7 Fig — (A) Time–series plot of monthly dengue cases with monthly average NDVI values (2012–2019), (B-C) Association of dengue incidence with different ranges of NDVI values. (TIF) [file pntd.0010859.s008.tif]

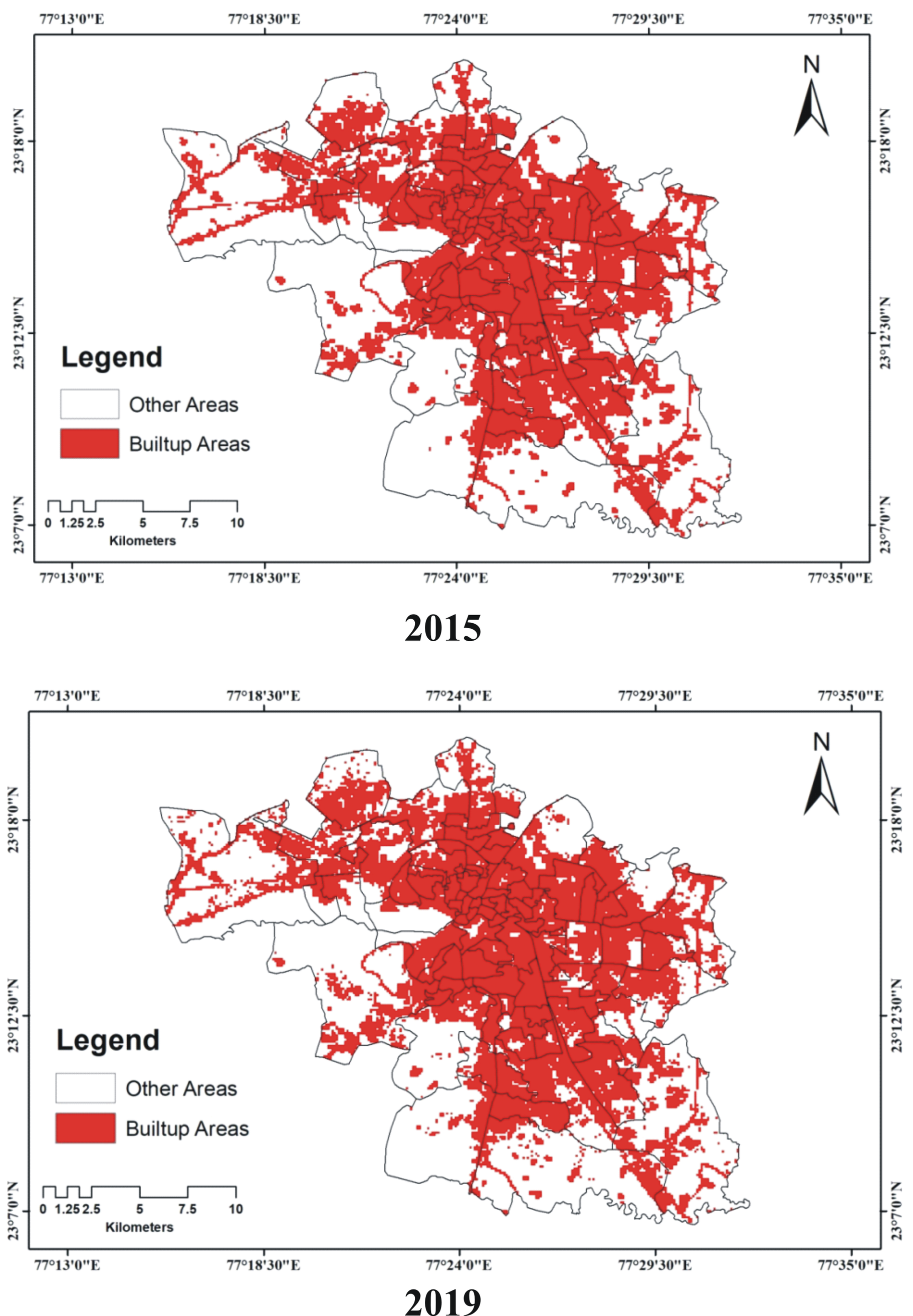

Supplement: S8 Fig — (TIF) [file pntd.0010859.s009.tif]
