# Supplementary material for: An assessment of remotely sensed environmental variables on Dengue epidemiology in Central India
Source: PLoS Negl Trop Dis. 2022 Oct 17;16(10):e0010859. doi: 10.1371/journal.pntd.0010859 (PMC9612820; doi:10.1371/journal.pntd.0010859)
Supplement: S1 Table — (DOCX) [file pntd.0010859.s010.docx]

**S1 Table: Details of the satellite images used for land use land cover (LULC) classification of Bhopal city.**

| Satelite | Sensor | Resolution | Bands used | Path/Row | Date of acquisition | Source |
| --- | --- | --- | --- | --- | --- | --- |
| Landsat 8 | OLI (Operational Land Imager) | 30m | 5,4,3 | 145/44 | O7-04-2014 | https://earthexplorer.usgs.gov/ |
| Landsat 8 | OLI (Operational Land Imager) | 30m | 5,4,3 | 145/44 | 07-04-2020 | https://earthexplorer.usgs.gov/ |
