# Supplementary material for: An assessment of remotely sensed environmental variables on Dengue epidemiology in Central India
Source: PLoS Negl Trop Dis. 2022 Oct 17;16(10):e0010859. doi: 10.1371/journal.pntd.0010859 (PMC9612820; doi:10.1371/journal.pntd.0010859)
Supplement: S2 Table — (DOCX) [file pntd.0010859.s011.docx]

**S2 Table: Descriptive statistics of weekly values of weather variables from 2012 to 2019 in Bhopal city.**

| **Variables** | **2012** | | | | | | | **2013** | | | | | | | **2014** | | | | | | |
| --- | --- | --- | --- | --- | --- | --- | --- | --- | --- | --- | --- | --- | --- | --- | --- | --- | --- | --- | --- | --- | --- |
|  | **Mean** | **Min** | **Max** | **SD** | **Percentile** | | | **Mean** | **Min** | **Max** | **SD** | **Percentile** | | | **Mean** | **Min** | **Max** | **SD** | **Percentile** | | |
|  |  |  |  |  | **25%** | **50%** | **75%** |  |  |  |  | **25%** | **50%** | **75%** |  |  |  |  | **25%** | **50%** | **75%** |
| **Mean Temp** | 25.56 | 16.24 | 36.4 | 5.28 | 21.01 | 25.46 | 30.23 | 25.24 | 16.54 | 36.68 | 5.02 | 20.84 | 25.84 | 27.66 | 25.79 | 14.82 | 37.78 | 5.99 | 21.11 | 25.43 | 30.8 |
| **Max Temp** | 32.51 | 25.07 | 43.87 | 5.16 | 28.73 | 30.1 | 37.55 | 31.66 | 25.01 | 44.71 | 5.1 | 28.46 | 29.41 | 35.09 | 32.42 | 22.82 | 45.49 | 6.03 | 28.66 | 29.92 | 38.99 |
| **Min Temp** | 18.62 | 7.29 | 28.94 | 6.45 | 12.11 | 19.92 | 23.83 | 18.82 | 6.73 | 28.97 | 6.11 | 12.51 | 21.41 | 23.48 | 19.15 | 6.16 | 30.08 | 5.52 | 12.94 | 20.29 | 23.99 |
| **DTR** | 13.88 | 4.27 | 21.72 | 4.97 | 10.09 | 15.06 | 17.85 | 12.84 | 4.21 | 21.37 | 5.09 | 7.38 | 14.74 | 17.41 | 13.26 | 4.77 | 20.25 | 4.18 | 10.58 | 14.27 | 16.61 |
| **Rainfall (mm)** | 3.1 | 0 | 39.73 | 7.09 | 0 | 0.1 | 1.75 | 4.3 | 0 | 37.22 | 2.14 | 0.002 | 0.447 | 4.28 | 2.73 | 0 | 21.72 | 5.11 | 0.02 | 0.33 | 2.73 |
| **RH (%)** | 47.8 | 9.47 | 91.78 | 26.13 | 23.11 | 44.19 | 70.93 | 54.99 | 15.78 | 92.87 | 26.69 | 26.59 | 54.16 | 84 | 51.67 | 15.08 | 90.55 | 23.45 | 34.6 | 49.12 | 73.32 |
|  |  |  |  |  |  |  |  |  |  |  |  |  |  |  |  |  |  |  |  |  |  |
| **Variables** | **2015** | | | | | | | **2016** | | | | | | | **2017** | | | | | | |
|  | **Mean** | **Min** | **Max** | **SD** | **Percentile** | | | **Mean** | **Min** | **Max** | **SD** | **Percentile** | | | **Mean** | **Min** | **Max** | **SD** | **Percentile** | | |
|  |  |  |  |  | **25%** | **50%** | **75%** |  |  |  |  | **25%** | **50%** | **75%** |  |  |  |  | **25%** | **50%** | **75%** |
| **Mean Temp** | 25.71 | 15.69 | 36.67 | 5.48 | 23.11 | 25.85 | 28.82 | 26.02 | 16.29 | 37.68 | 5.37 | 21.02 | 25.56 | 29.39 | 26.25 | 16.1 | 36.65 | 5.17 | 21.83 | 26.12 | 30.04 |
| **Max Temp** | 32.36 | 20.09 | 44.72 | 5.61 | 29.05 | 31.4 | 34.29 | 32.69 | 23.79 | 45.86 | 5.61 | 28.82 | 29.69 | 37.46 | 33.18 | 24.5 | 44.04 | 5.17 | 29.71 | 31.25 | 36.93 |
| **Min Temp** | 19.06 | 7.15 | 29.01 | 6.13 | 13.98 | 20.26 | 23.81 | 19.35 | 8.8 | 29.79 | 6.24 | 12.85 | 21.2 | 23.89 | 19.33 | 7.7 | 29.49 | 6.02 | 13.87 | 21.8 | 23.77 |
| **DTR** | 13.3 | 4.65 | 19.3 | 4.22 | 9.57 | 13.84 | 17.01 | 13.34 | 4.28 | 20.31 | 5.07 | 7.51 | 15.25 | 17.64 | 13.84 | 6.13 | 20.26 | 4.4 | 9.5 | 15.15 | 17.43 |
| **Rainfall (mm)** | 3.65 | 0 | 45.48 | 7.74 | 0.003 | 0.28 | 3.09 | 4.18 | 0 | 55.44 | 9.5 | 0 | 0.03 | 3.63 | 2.33 | 0 | 17.06 | 4.52 | 0 | 0.02 | 2.46 |
| **RH (%)** | 51.43 | 14.21 | 89.86 | 23.91 | 28.61 | 49.76 | 71.85 | 49.3 | 13.55 | 92.28 | 27.63 | 26.31 | 40.99 | 84.49 | 45.92 | 7.85 | 85.96 | 25.32 | 21.58 | 41.18 | 72.32 |
|  |  |  |  |  |  |  |  |  |  |  |  |  |  |  |  |  |  |  |  |  |  |
| **Variables** | **2018** | | | | | | | **2019** | | | | | | | **2012-2019** | | | | | | |
|  | **Mean** | **Min** | **Max** | **SD** | **Percentile** | | | **Mean** | **Min** | **Max** | **SD** | **Percentile** | | | **Mean** | **Min** | **Max** | **SD** | **Percentile** | | |
|  |  |  |  |  | **25%** | **50%** | **75%** |  |  |  |  | **25%** | **50%** | **75%** |  |  |  |  | **25%** | **50%** | **75%** |
| **Mean Temp** | 26.14 | 16.06 | 36.88 | 5.43 | 22.38 | 25.45 | 29.49 | 25.48 | 12.31 | 38.65 | 6.24 | 20.32 | 25.65 | 29.56 | 25.9 | 11.54 | 39.29 | 5.53 | 21.94 | 25.72 | 29.19 |
| **Max Temp** | 33.02 | 26.27 | 44.6 | 5.58 | 28.95 | 31.23 | 37.21 | 31.65 | 20.29 | 46.06 | 6.59 | 27.51 | 28.73 | 37.32 | 32.52 | 17.56 | 47.52 | 5.73 | 28.59 | 30.61 | 36.48 |
| **Min Temp** | 19.27 | 5.67 | 29.51 | 6.19 | 13.97 | 20.42 | 24.09 | 19.31 | 4.34 | 31.26 | 6.86 | 13.36 | 21.07 | 24.05 | 19.27 | 2.31 | 33.21 | 6.37 | 13.87 | 20.8 | 23.96 |
| **DTR** | 13.74 | 4.74 | 20.79 | 4.6 | 9.56 | 15.57 | 17.03 | 12.34 | 3.69 | 18.99 | 5.04 | 7.69 | 12.32 | 17.42 | 13.26 | 1.78 | 23.74 | 4.95 | 8.86 | 14.27 | 17.4 |
| **Rainfall (mm)** | 2.55 | 0 | 19 | 4.98 | 0 | 0.014 | 2.94 | 4.98 | 0 | 44.24 | 9.46 | 0.003 | 0.2 | 4.31 | 3.52 | 0 | 132.3 | 10.21 | 0 | 0 | 1.32 |
| **RH (%)** | 45.38 | 10.46 | 91.23 | 27.05 | 22.08 | 42.33 | 70.17 | 54.19 | 13.08 | 94.59 | 29.98 | 23.43 | 65.29 | 81.77 | 50.23 | 5.32 | 95.43 | 26.92 | 24.35 | 46.86 | 78.07 |
