# Supplementary material for: An assessment of remotely sensed environmental variables on Dengue epidemiology in Central India
Source: PLoS Negl Trop Dis. 2022 Oct 17;16(10):e0010859. doi: 10.1371/journal.pntd.0010859 (PMC9612820; doi:10.1371/journal.pntd.0010859)
Supplement: S3 Table — (DOCX) [file pntd.0010859.s012.docx]

**S3 Table: Cross-correlation analysis between dengue cases and monthly NDVI at the different lag periods in Bhopal City**

| Lag month | *r*^2^ value | Significance |
| --- | --- | --- |
| 0 | 0.303 | p<0.0001 |
| 1 | 0.339 | p<0.0001 |
| 2 | 0.092 | p<0.0001 |
| 3 | 0.003 | Non-significant |
| 4 | 0.118 | p<0.01 |
| 5 | 0.198 | p<0.0001 |
| 6 | 0.179 | p<0.0001 |
| 7 | 0.081 | p<0.001 |
| 8 | 0.004 | Non-significant |
