# Supplementary material for: An assessment of remotely sensed environmental variables on Dengue epidemiology in Central India
Source: PLoS Negl Trop Dis. 2022 Oct 17;16(10):e0010859. doi: 10.1371/journal.pntd.0010859 (PMC9612820; doi:10.1371/journal.pntd.0010859)
Supplement: S4 Table — (DOCX) [file pntd.0010859.s013.docx]

**S4 Table: Changes in NDVI index in high endemic wards, low endemic wards and overall Bhopal city from 2012-2019**

| **Ward number** | | **Average values of NDVI** | | | | | | | |
| --- | --- | --- | --- | --- | --- | --- | --- | --- | --- |
|  |  | **2012** | **2013** | **2014** | **2015** | **2016** | **2017** | **2018** | **2019** |
| **Wards with high incidence of dengue** | **31** | 0.46 | 0.48 | 0.48 | 0.47 | 0.47 | 0.47 | 0.45 | 0.47 |
|  | **32** | 0.34 | 0.37 | 0.38 | 0.36 | 0.35 | 0.37 | 0.34 | 0.35 |
|  | **44** | 0.24 | 0.26 | 0.26 | 0.25 | 0.24 | 0.25 | 0.26 | 0.28 |
|  | **45** | 0.32 | 0.35 | 0.36 | 0.35 | 0.34 | 0.35 | 0.33 | 0.36 |
|  | **46** | 0.43 | 0.46 | 0.48 | 0.45 | 0.44 | 0.46 | 0.43 | 0.47 |
|  | **49** | 0.31 | 0.34 | 0.33 | 0.33 | 0.33 | 0.34 | 0.32 | 0.37 |
|  | **53** | 0.37 | 0.39 | 0.41 | 0.40 | 0.41 | 0.41 | 0.41 | 0.43 |
|  | **54** | 0.39 | 0.42 | 0.44 | 0.42 | 0.42 | 0.43 | 0.42 | 0.46 |
|  | **56** | 0.44 | 0.48 | 0.52 | 0.48 | 0.50 | 0.52 | 0.50 | 0.54 |
|  | **57** | 0.42 | 0.47 | 0.50 | 0.47 | 0.46 | 0.47 | 0.45 | 0.50 |
|  | **58** | 0.36 | 0.40 | 0.42 | 0.40 | 0.41 | 0.39 | 0.38 | 0.43 |
|  | **64** | 0.28 | 0.31 | 0.37 | 0.31 | 0.34 | 0.39 | 0.34 | 0.37 |
|  | **67** | 0.26 | 0.30 | 0.30 | 0.29 | 0.29 | 0.30 | 0.29 | 0.31 |
|  | **80** | 0.35 | 0.36 | 0.36 | 0.37 | 0.35 | 0.36 | 0.38 | 0.40 |
|  | **82** | 0.29 | 0.32 | 0.33 | 0.33 | 0.33 | 0.35 | 0.33 | 0.36 |
|  | **Average** | *0.35* | *0.38* | *0.40* | *0.38* | *0.38* | *0.39* | *0.38* | *0.41* |
| **Wards with low incidence of dengue** | **1** | 0.41 | 0.42 | 0.45 | 0.40 | 0.45 | 0.48 | 0.46 | 0.53 |
|  | **3** | 0.43 | 0.47 | 0.50 | 0.45 | 0.48 | 0.52 | 0.50 | 0.51 |
|  | **4** | 0.39 | 0.42 | 0.38 | 0.43 | 0.32 | 0.43 | 0.37 | 0.31 |
|  | **5** | 0.39 | 0.43 | 0.41 | 0.45 | 0.39 | 0.43 | 0.40 | 0.41 |
|  | **10** | 0.29 | 0.31 | 0.31 | 0.31 | 0.32 | 0.33 | 0.33 | 0.38 |
|  | **11** | 0.34 | 0.38 | 0.40 | 0.36 | 0.39 | 0.39 | 0.37 | 0.46 |
|  | **14** | 0.20 | 0.21 | 0.21 | 0.21 | 0.23 | 0.23 | 0.22 | 0.27 |
|  | **15** | 0.34 | 0.37 | 0.37 | 0.34 | 0.32 | 0.36 | 0.34 | 0.36 |
|  | **16** | 0.32 | 0.37 | 0.39 | 0.36 | 0.39 | 0.38 | 0.36 | 0.40 |
|  | **20** | 0.27 | 0.32 | 0.33 | 0.34 | 0.34 | 0.35 | 0.32 | 0.35 |
|  | **23** | 0.18 | 0.19 | 0.18 | 0.18 | 0.19 | 0.20 | 0.20 | 0.22 |
|  | **47** | 0.48 | 0.54 | 0.55 | 0.53 | 0.53 | 0.54 | 0.53 | 0.55 |
|  | **72** | 0.36 | 0.40 | 0.43 | 0.38 | 0.43 | 0.45 | 0.42 | 0.47 |
|  | **76** | 0.36 | 0.38 | 0.39 | 0.36 | 0.39 | 0.41 | 0.38 | 0.42 |
|  | **77** | 0.27 | 0.31 | 0.31 | 0.30 | 0.32 | 0.32 | 0.32 | 0.34 |
|  | **Average** | *0.34* | *0.37* | *0.38* | *0.36* | *0.36* | *0.39* | *0.37* | *0.40* |
| **Bhopal NDVI** | | *0.29* | *0.31* | *0.33* | *0.32* | *0.31* | *0.31* | *0.29* | *0.32* |
| **Maximum NDVI** | | 0.49 | 0.52 | 0.54 | 0.53 | 0.51 | 0.53 | 0.51 | 0.54 |
| **Minimum NDVI** | | 0.14 | 0.15 | 0.15 | 0.15 | 0.15 | 0.15 | 0.15 | 0.15 |
